# Thoughts on the Etiology of Cherubism

**DOI:** 10.3390/jcm13072082

**Published:** 2024-04-03

**Authors:** Peter Hyckel, Thomas Liehr

**Affiliations:** Jena University Hospital, Friedrich Schiller University, Institute of Human Genetics, 07747 Jena, Germany; peter.hyckel@web.de

**Keywords:** cherubism, WNT/MSX1 pathway, defective tooth development

## Abstract

Cherubism is nowadays classified as an autoimmune disease and was first described in 1933. Although suspected at that time to be the result of defective tooth development, it was primarily classified as a bone disease caused by a mutation in the *SH3BP2* gene. Despite a knock-in mouse model, phenotypic signs in the jaw area were not reproducible in this model. The features of classical cherubism can be attributed to a disturbed formation of the dental placode of the second molar. Since 2019, it has become clear that inhibition of the WNT pathway leads to the accumulation of SH3BP2 via tankyrase inhibition. As the dental placode is triggered via WNT (in epithelia) and MSX1 (in mesenchyme), aplasia of the second and third molars occurs due to a block in the WNT pathway. The mesenchymal part, which occurs prior to the body plan regulation of the WNT/MSX1 pathway, remains unaffected and provides the substrate for the giant cell granuloma. Considering macrophage polarization and the role of the extracellular matrix in general, cherubism is situated in the field of tension between autoimmune diseases and cancer. In this sense, we see the cause of cherubism in a WNT-related dysregulation, which can be proven postnatally in the neural crest-related tooth development of the replacement tooth ridge, both genotypically and phenotypically.

## 1. Background

Cherubism is a rare disease of the jawbones and the disease is self-limiting, but its pathogenesis is still under discussion. The majority of publications regard cherubism as a kind of a general bone disease. However, the author of the first description [1] already suspected a dental cause, i.e., exactly the putative cause that we pointed out in 2005 [2]. Later on, in 2018, we indicated that a sound diagnosis of cherubism must take into account the clinical course of the disease as well as the morphology and molecular biology [3]. Here, the metabolic factors derived from the neural crest are of particular importance, which refers to the results of our work from 2005 [2], in which a dental pathogenesis was prioritized. Based on a connection with the development of the second and third molars, cherubism could be understood as a genetically determined change in tooth development. In this context, the impaired PTHrP–PTHrP receptor interaction induced by mutations in the SH3BP2 gene was discussed. Accordingly, the temporal and spatial expression of clinical symptoms can then be explained by an interaction of SH3BP2-dependent signal transduction pathways with jaw morphogenesis (e.g., homeobox gene MSX1). In cherubism, due to the disease-related lack of the cap phase development of second and third molars, there is no spatial placement or normal tooth development. Instead, a cystic/fibrous tissue with giant cells develops [3].

**Figure 1:**
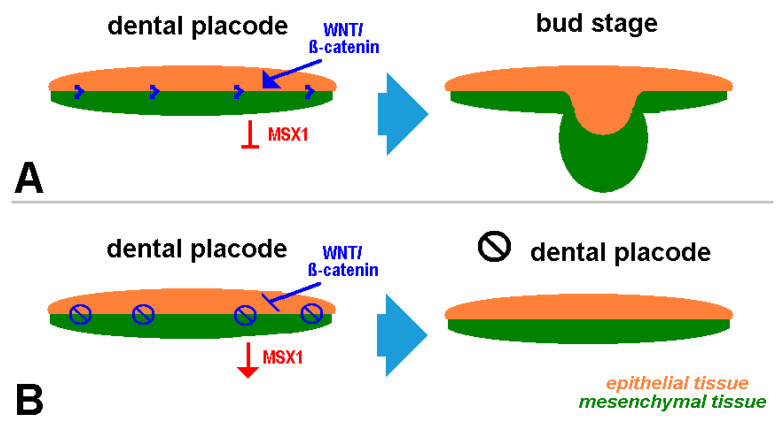
Schematic depiction of dental placode development: normal (**A**) and disturbed (**B**).

According to Pispa and Thesleff [4], ectodermal organs like teeth or hair have the following feature in common: they derive from neighboring layers of epithelial and mesenchymal tissues (see also Figure 1). Tooth development is linked to the corresponding gene expression in the epithelium and mesenchyme from dental lamina to the bell stage [5]. For the understanding etiology of cherubism, only the transition from the dental lamina to the bud stage needs to be considered. During dentogenesis/incipient tooth development, the epithelium (which is, among other factors, triggered by WNT) is drawn into the jawbone marrow (Figure 1); the latter is mediated by mesenchymal neural crest genes (e.g., *MSX1* and *PAX9*). MSX1 plays a key role in the transition from the bud to cap stage. On the other hand, epithelial WNT signaling activity is responsible for the MSX1-dependent odontogenic pathway during early tooth morphogenesis [6,7].

While MSX1 expression is the leading factor in normal tooth development, the WNT signaling pathway (Figure 1) controls it. If the latter is disturbed, the bud stage is not realized, but parts of the dental lamina persist in the jaw mesenchyme with continued MSX1 expression. The interactions described by us in 2005 [2] during tooth development according to Bei and Maas [8] are still the standard today.

## 2. Origin of the Name, Symptoms, and Pathogenesis of Cherubism

The decisive factor of applying the name cherubism in the first description from 1933 was the phenotype described as: “The full round cheeks and the upward cast of the eyes give the children a peculiarly grotesque, cherubic appearance” [3]. This may have been a comparison with the trombone angels, or cherubs, who have an infantile appearance and turn their eyes heavenwards when blowing; in other words, a derivation from Christian culture that cannot be understood in this way everywhere. Jones also reports on a family with children aged six, five, and four years in whom symptoms were detectable in the upper and lower jaw. The teeth there were irregularly arranged, and many were missing. The alveolar ridges were extremely wide and lead to a narrow V-shaped palate in the upper jaw. The name of the clinical picture was coined by Jones on the basis of the clinical signs of cherubism [9]. The deciduous tooth position was irregular [10], which was interpreted as excessive deciduous tooth resorption [11].

Biernat [12] aptly describes the pathogenesis in her dissertation (written in German and translated and adapted here): Tooth development was suspected early on as a trigger for cherubism [11]. The time of manifestation of the disease and the marked involvement of the maxilla and mandible are indications of this possibility. It has been hypothesized that the growing tooth germs are a triggering stimulus to the jawbones and cause a pathological (excessive osteolytic) reaction in the tissue [11]. The second and third molars are the only teeth there that are not yet fully developed at birth. As the mineralization of these teeth begins at the age of two to three years, a connection with the development of the disease is possible [2]. Remission and the “skyward gaze” can also be explained by this. These connections are explained via tooth follicle formation using molecular genetic interactions of the BMP, FGF, WNT, and Hh gene families. The transition from the lamina to the bud stage, which is characterized by the interaction of WNT/MSX1 and PAX9, was also of particular interest to other researchers [13].

## 3. Molecular Biology

In 1999, the gene responsible for cherubism was mapped to chromosome 4p16.3 [14,15]. In 2001, Ueki et al. [16] succeeded at identifying a disease-causing point mutation in the *SH3BP2* gene. Accodingly, cherubism has been characterized as a hereditary bone disease that affects the upper and lower jaw and leads to dislocations latest by then. The molecular genetic connection with the dental system that we propagated [2] was at that time dismissed as hypothetical in the mainstream literature, and everything that showed cystic or granulomatous changes in the jaw was defined as cherubism because *SH3BP2* mutations were detected.

In 1998, Kalantar Motamedi [17] introduced a graded system (5 stages) under the name of cherubism, among others, in order to achieve higher case numbers for representative studies. Exclusively, the classic course, as described by Jones [1], is classified as grade 5 there.

Ueki and Reichenberger, as the most important researchers in cherubism field, have defined the disease via the myeloid progenitor cell (hyperactive macrophages/autoimmune response) [16,18]. They attempted to prove the pathogenesis as a bone disease via a knock-in mouse model of *SH3BP2*, which has so far been insufficiently successful. A possible dentogenesis of cherubism was only considered by Reichenberger et al. [18] (albeit as a tooth eruption problem) and was not otherwise considered. The number of studies on cherubism has risen sharply since 2001, as soon as the disease had been connected to a certain gene. To date, around 500 cases classified as cherubism have been reported [19]. There are almost 600 corresponding publications in PubMed [20].

Concerning pathways, Levaot et al. [21] referred to the proven connection between the SH3BP2 protein and tankyrase, and Mukai et al. [22] have impressively demonstrated the connection of SH3BP2 accumulation, WNT inhibition, and loss of bones/reduction in bone formation.

In 2005, our working group had substantiated dental genesis due to overexpression of mesenchymal MSX1 [2]. According to Medio et al. [23], imbalances in WNT signaling and MSX1 activity lead to defects in craniofacial development (related to clefts). Concerning tooth development at the bell stage, Feng et al. [24] concluded that MSX1 maintains cell proliferation by inhibiting the WNT signaling pathway while preventing odontoblast differentiation. The process of tooth development from the oral epithelium is characterized by MSX1 and PAX9, both being mesenchymal markers that draw the epithelial component into the jaw stroma. This process is regulated by WNT10a/b. If WNT fails, tooth aplasia occurs. This can be a one-time process, not familial. If the WNT/ß-catenin signaling pathway is mutated, like in cherubism, teeth 1 to 6 in all quadrants are still laid down properly in the fetal phase, as are all organs. Because the dental follicles of the replacement dental ridge of teeth 7 and 8 only develop postnatally, the clinically visible effect only occurs then; WNT inhibition leads to SH3BP2 accumulation [22]. This is the binding process for cherubism as a familial disease, but it is not superselective.

The fundamental question is whether this may still occur according to fetal developmental signaling pathways following the body plan, although showing up postnatally. In the following, we will attempt to answer this question using data from the literature concerning the immunology (CD 163) and parameters of the extracellular matrix (ECM), such as tenascin or fibronectin. Basically, we assume that autoimmune reactivity corresponds to an alternative macrophage polarization (M2), similar to neuromuscular sarcoidosis [25].

## 4. Relation to the Extracellular Matrix and Immunology

The results of Kwack et al. [26] suggest that mandibular bone marrow-derived cells show consistent deficits in myeloid differentiation, including significantly fewer myeloid-derived suppressor cells (MDSCs); however, these showed increased immunosuppressive activity compared with long bones. It is also typical for skeletal diseases more likely affecting alveolar bones as in hyperparathyroid jaw tumor syndrome, cherubism, and osteonecrosis associated with antiresorptive therapeutics [26]. According to Friedrich et al. [27], CD163 is upregulated in giant cell granuloma in cherubism, which Wehrhan et al. [28] described as an M2 marker in metastatic squamous cell carcinoma as well. This is not a contradiction but illustrates the connection between autoimmune disease and advanced cancer. Furthermore, ß-catenin was not expressed in cancer, which illustrates the connection with the impaired WNT/ß-catenin signaling pathway. Since, according to Gupta et al. [29], tenascin C (TNC) and fibronectin are also expressed in giant cell granuloma, this parallels ECM in oral squamous cell carcinoma [30]. Furthermore, Haas et al. [31] have described laminin-5 expression both in cancer and in blistering dermatoses.

Tenascin C (TNC), having many binding partners like fibronectin, collagen, fibrillin-2, and others, has a major role in cell proliferation and migration. TNC is mainly expressed in embryonal tissues, however, it is present again in adult tissues during inflammation, wound healing, cancer, and other pathological conditions [32].

## 5. Clinical Signs of Cherubism Related to WNT/ß-Catenin/MSX1 Expression

The characterization of the *SH3BP2* mutations that leads to WNT inhibition, in terms of loss of control [33], was a breakthrough in understanding cherubism. What still remains unanswered is in which way such single gene mutations lead to problems primarily restricted to the jaw and/or why there is a phenotype regression after puberty [33]. The jaw association is defined by the neural crest reference. Nassif et al. [34] proposed MSX1 overexpression as a modeling factor for the membranous jawbone. In line with this, Houpis et al. [35] reported that PTHrP and MSX1 are expressed in the giant cell granuloma in the jaw. Because tooth formation is not realized and PTHrP is expressed (in PTHrP knockout mice) for tooth eruption while the substrate is not created, osteoclasts with TRAP activity [36] and uncontrolled osteoclastogenesis occur at the cortical bone of the jawbone, which may contribute to the formation of giant cell granuloma. Liu et al. [37] have highlighted the fate of the dental placement when the WNT/ß-catenin pathway is dysregulated. Parts of the epithelial remnants retracted by MSX remain intact in this way.

## 6. Relation to the Periodontium

Because cherubism is a disease that only becomes phenotypically relevant after the second year of life, the deciduous teeth are initially normally developed but then show irregularities with premature resorption. Spodzieja and Olczak-Kowalczyk [38] also consider the premature loss of deciduous teeth to be a symptom of a systemic disease in cherubism. However, the genetic dysregulation only has relevant effects in postnatally developed tooth germs.

Tooth agenesis occurs due to the failure of the teeth to develop from the replacement-tooth ridge as a result of the misexpression of embryonic transcription factors, e.g., MSX1 [39]. On the other hand, Kapadia et al. [40] found tooth agenesis in cases without *PAX9* and *MSX1* gene mutations. Overall, it appears that not only mutation but also the overexpression of genes may lead to tooth loss, which does not mean that all periodontal structures are lost. The periodontium consists of the interaction of cementum and alveolar bone [41]. The basis of this interaction is the Sharpey fibers involved in the tooth/bone interface and their molecular mechanisms, as described by Fleischmannova et al. [42]. As the counterpart is missing in this interaction, a periodontium is not formed but the osseous/osteoclastic/fibrous side overdevelops. The periodontal stem cells undergo osteogenic differentiation [43] but retain their odontogenic potential [44]. These osteogenically differentiated periodontal stem cells have low immunogenicity [43] with expression of CD163 [45], suggesting M2 polarization. This could be due to expression according to quasi-fetal conditions from the body plan. **In our view, this is the basis of the developing disorganized fibrous replacement structures in the dental distal neural crest area.**

Regarding central odontogenic fibroma or giant cell granuloma, Tosios et al. [46] noted that, here, reduced cellular fibroblast proliferation and accordingly variable odontogenic epithelial component are observed. Central giant cell lesions are thus osteolytic fibroblastic proliferations characterized by osteoclast-like multinucleated giant cells, which are zones of collagen fibers with a vertebral pattern and cystic changes that can be interpreted as aneurysmal bone cysts. Increased MSX1 expression is of the same magnitude as that required for tooth development. 

Accordingly, the debate as to whether the central cemento-ossifying fibroma is of osteogenic or dental origin appears to be identical. Although classified as osteogenic by the WHO, it is now attributed to a genesis recruited from the periodontal ligament [47]. Phenotypically, it may be very similar to the findings in cherubism.

It is interesting to note that Friedrich et al. [45] found in neurofibromatosis type 1 (NF1) that dentists or maxillofacial surgeons may be the first to detect aplasia of the second lower molars or other numerical aberrations in the dentition of children with NF1. In classical cherubism, aplasia of the replacement dentition is also a leading symptom. Because activation of the WNT signaling pathway is a hallmark of NF1 [48], this finding does not seem surprising; however, **in our opinion, this can be interpreted as a false expression of the remnants of the dental lamina**.

Because PTHrP is required for tooth eruption and, according to the body plan, the cortical bone is rounded up aimless, PTHrP is switched off after complete tooth development and regresses. Jones has already pointed out that even the teeth that are designed for development do not correspond to the dental arch, and many are missing [1]. We have already commented on the missing teeth above.

The WNT-sensitive signaling pathway comprises crucial molecular cascades for cell metabolism and is essential for bone/cementum remodeling in response to orthodontic forces [49]. In the case of WNT/ß-catenin signaling dysfunction, disordered placement can be expected in teeth that have not yet erupted. Hermanns et al. [50] have reported on the interaction between canonical WNT and SHH in the stages of mouse tooth formation, starting from the formation of the dental placode to the fully formed adult tooth. In classical cherubism, the minimization of the inhibition zone leads to incorrect tooth placement, similar to the teeth sliding past each other, which logically results in a widening of the alveolar process. This circumstance simulates the symptom of a high palate (V-palate). A possible role of TNC in the etiopathogenesis of central giant cell lesions in the jaw is pointed out by Tobon-Arroyave et al. [51]. TNC mRNA is expressed in cranial neural crest cells, in some placodal derivatives, and in discrete domains of the embryonic zebrafish brain [52]. The dental epithelium induces the expression of TNC in the early dental mesenchyme in mice [53]. From this point of view, it can be deduced that the central giant cell granuloma is a product of failed tooth development with the placode remnants.

**Figure 2:**
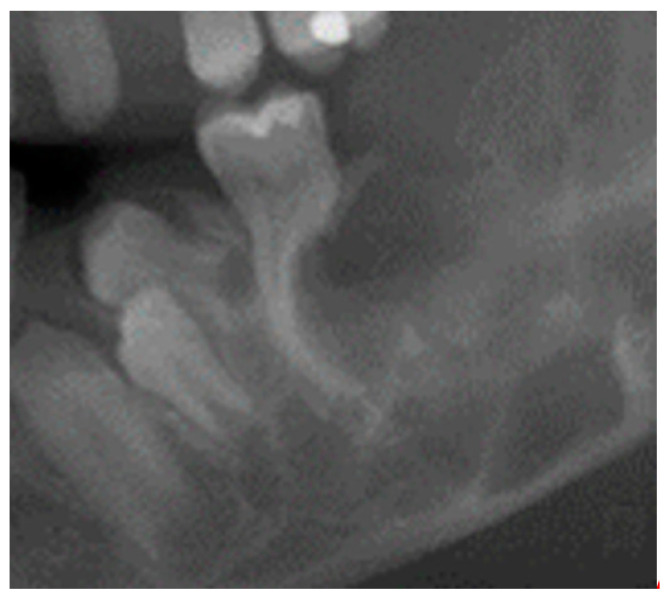
An X-ray showing root resorption in a cherubism patient after contact with osteoprotegerin (OPG).

A radiologic sign of cherubism is root resorption (e.g., on six-year molars) [2]. Although the distal root was formed, it is resorbed in contact with osteoprotegerin = OPG [54] via RANKL = receptor activator of NF-κB ligand (possibly involving MSX1) due to the lack of WNT/ß-catenin signaling. This finding was also described from a radiological perspective (MRI = magnetic resonance imaging) in the same patient, as shown in Figure 2 [55].

One clinical sign that we have included here is the integrity of the articular process. This is important because Seward and Hankey [56] considered the involvement of the condyle, or not, as a measure of severity. We believe that the condyle, like the diastema, is SHH-triggered. The proximity to the articular cartilage suggests this. The indolent clinical swelling of the lymph nodes is also a symptom of classic cherubism. Often assessed as lymphadenopathy without an inflammatory cause, Chen Wongworawat et al. [57] described the lymph nodes as hemosiderin-laden macrophages and basophilic laminated concretions. We interpret this as a reaction of hyperactive macrophages in the sense of fibrotic changes.

## 7. Summarized Evaluation from a Semantic, Animal Experiment, and Study Perspective

From the above explanations, the entire symptomatology of classic familial cherubism can be adequately explained. In this sense, from a “semantic point of view”, we should only speak of cherubism if the classic criteria are fulfilled, or otherwise the term “cherubism like” should be used. With regard to the age of the patient when the clinical picture is first perceived, there are variations between 2 and 8 years. Chrcanovic et al. [19] stated 5.6 ± 3.8 years to be the range for the first occurrence. This is approximately the mean value between the beginning of the development of the dental follicles of the replacement dental ridge. Here, 2–3 years applies to classic cherubism and 8–9 years to non-familial cherubism. Chrcanovic et al. [19] therefore recognized the symptom onset at 2–3 years as the basic context of our 2005 work [2] and concluded that “interactions between SH3BP2-dependent signal transduction pathways and mechanisms involved in tooth development and jaw morphogenesis have been proposed to explain the rate of molar agenesis in patients with cherubism. The fact that the clinical course of cherubism coincides with the developmental period of the second and third molars supports this hypothesis”.

Fuiji et al. [58] and co-authors including Ueki and Reichenberger have stated that the mechanism for activation of CBM lesions are still unclear despite the availability of CBM knock-in (KI) mouse models (Sh3bp2 KI/KI); the latter do not show the phenotype of expansive jawbones. 

The following errors of reasoning having always played a decisive role in all appreciation of the aforementioned works:

- Mice have a different dental development and signaling pathways than humans: while all teeth are laid down prenatally in mice, in humans, the formation of the second and third molars only occurs postnatally via the replacement dentition, parallel to the formation of the first symptoms of classic cherubism.
- While the mandible originates from the cranial neural crest, the bones of the limbs are of mesodermal origin [59]. Because tubular bone is formed mesenchymally, “neural crest cells (NCCs)” derive from dorsal side of the neural tube; they proliferate rapidly and migrate along predetermined, well-characterized routes into developing frontonasal plate and gill arches. This is the reason for the contribution of NCCs to many craniofacial structures [60].
- Accordingly, the alveolar process has a different immunology than long bones in the sense that, under pathologic conditions, the differentiation of immature myeloid cells generated in the bone marrow is partially blocked into mature myeloid cells. This results in MDSCs [25]. The mechanism of the tankyrase inhibition of PARsylation-mediated ubiquitylation can be connected with cherubism [61].

**Figure 3:**
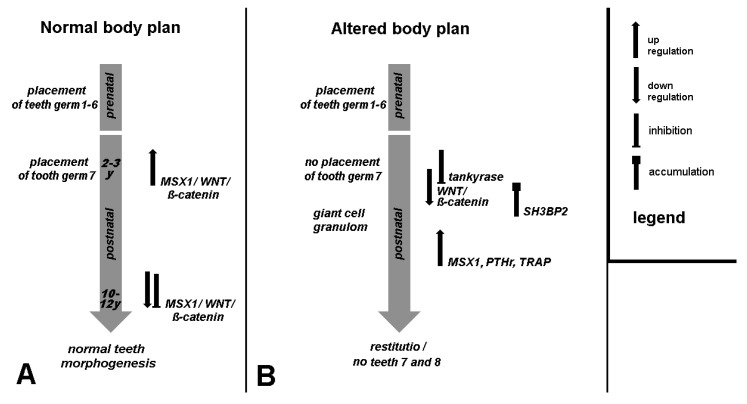
Normal, and in case of cherubism (altered), disturbed tooth germ development; the scheme shows the establishment of permanent teeth in connection with the replacement toothbar. On the right the legend for the used symbols is provided concerning regulation of involved genes and proteins.

It is therefore unlikely that a further increase in the number of cases (meta-studies) will provide more reliable insights into the pathogenesis of cherubism. For example, Schreuder et al. [62] spoke of a third, less probable explanation via dental embryology [2], without justifying the restriction, and classified it as RASopathy. Thus, we adapted the flowchart we published in 2005 to the current findings (Figure 3).

## 8. Therapy

It is generally recognized that “wait and see” is a tried and tested procedure for cherubism due to self-limitation. The body plan for the tooth development of the replacement dentition takes many years, which requires patience from patients and practitioners. 

Cailleaux et al. [63] have reported on three types of drugs used to treat cherubism: calcitonin, immunomodulators, and antiresorptive agents (calcitonin, denosumab, bisphosphonates, anti-TNFα, tacrolimus, imatinib). Due to heterogeneity, an effect relationship could not be determined. On the other hand, Ricalde et al. [64] were able to demonstrate an impressive reduction of symptoms with imatinib. Tickenbrock et al. [65] were able to demonstrate activation of the WNT signaling pathway in acute myeloid leukemia under imatinib. The activation of WNT could therefore relativize the deficit in tooth morphogenesis. Erroneous WNT/β-catenin signaling activation has been connected with cancer induction via the overexpression of multidrug resistance (MDR)-related transporters [66]. This also shows a potential close relationship of tankyrases to oncology.

We can only comment on bisphosphonates with respect to osteonecrosis of the jaw, as Wehrhan et al. [67] described that MSX1 is inhibited by bisphosphonates, which is actually a cause-related therapy. On the other hand, Wehrhan et al. [68] found in BRONJ jawbone disease a bisphosphonate-mediated shift in macrophage polarization towards tissue-destructive M1 macrophages. The idea of [68] that M1 polarization could be associated with the regeneration of various tissues and could contribute to the antitumor effect of bisphosphonates was confirmed by Weber et al. [69] later. At the same time, a clear M2 polarization was attributed to the formation of dentogenic non-radicular cysts, which suggests a developmental origin [70].

Regarding the prescription of calcitonin to reduce the progression of cherubism, we agree with Meijer et al. [71] that this is a fundamental possibility. As the relationship of tankyrase to the WNT pathway is established, the importance of vitamin D as a prophylactic agent in cherubism is also logical. Lee et al. [72] recommended the simultaneous use of bisphosphonates and vitamin D in generally severe disease courses, which was associated with an additive reduction in mortality.

## 9. Conclusions

We assume that cherubism can be adequately explained as an autoimmune disease caused by dysregulation of the WNT/ß-catenin signaling pathway via a tooth development disorder. It is clear that the interaction of WNT, HOX, and hedgehog genes must be taken into account. In our view, this shows that the genetic dysregulation must be consistent with the pathogenesis. Both the molecular biological results from the ECM and macrophage polarization as an expression of immune regulation can provide support.

Cherubism is a rare disease. Because the WNT signaling pathway plays a prominent role in autoimmune diseases (fibrosis) and cancer by influencing tankyrase expression, much can be “learned” from it. This also applies to viral replication processes [73].

Because tankyrase inhibition also plays a role in severe COVID-19 progression [74], it is hypothetical but conceivable that cherubism-like symptoms (non-attachment of the second molar, central giant cell granuloma) are detectable until puberty in children who had such a progression between the second and third year of life.

## Data Availability

No new data were created or analyzed in this study. Data sharing is not applicable to this article.
